# International Congress on Tuberculosis

**Published:** 1907-12

**Authors:** 


### Abstracts and Selections.

### International Congress on Tuberculosis.

WASHINGTON, D. C., November 2, 1907.

Progress along all lines connected with the International Congress on Tuberculosis which is to take place in Washington from September 21 to October 12, 1908, was shown by the reports presented at a meeting of the Committee of Arrangements, held in New York, at the Associated Charities Building, Monday evening, October 28th. Dr. Lawrence F. Flick, of Philadelphia, Chairman of the committee, presided, and the other members present were: Dr. Joseph Walsh, Philadelphia, Secretary; Dr. John S. Fulton, Washington, Secretary General; Mr. William H. Baldwin, Washington; Dr. Hermann M. Biggs, New York; Dr. Frank Billings, Chicago; Mr. Edward T. Devine, New York; Mr. Livingston Farrand, New York; Dr. J. C. Greenway, Greenwich, Conn.; Dr. Chas. J. Hatfield, Philadelphia; Dr. Abraham Jacobi, New York; Dr. Alfred Meyer, Mrs. James E. Newcomb, New York; General Geo. M. Sternberg, Washington, and Dr. Wm. H. Welch, Baltimore.

The meeting was the first held since Dr. Flick's return from abroad, and his reports of his visits to the International Conference on Tuberculosis in Vienna and to the International Congress on Hygiene and Demography, at Berlin, were interesting features of the session. More than a thousand delegates were registered at Vienna, he said, and the gathering at Berlin was quite as large. The leading men in both associations are looking forward with a great deal of enthusiasm, Dr. Flick said, to the meeting in Washington next year, and about four hundred of the members of the foreign organizations may be expected to attend the Congress. The Conference selected this country as its place of meeting in 1908 just as the Congress did two years ago. The Conference and the Congress are two distinct organizations. The International Conference on Tuberculosis meets every year and keeps up a continuous organization with headquarters in Berlin. The International Congress on Tuberculosis meets only once in three years and does not maintain an international bureau in the intervals. Dr. Flick stated that at the International Conference interest centered especially in the time-worn subject of the routes of invasion for the tubercle bacillus. It seems to have been demonstrated that the disease may be contracted by both the respiratory route and the alimentary route. Though this does not make us much wiser in a practical way, still it is somewhat comforting to know that the respiratory route is less important than it was once thought to be. On the other hand, that information is compensated by the importance of the alimentary route.

In connection with his account of the progress made in the preliminary arrangements for the International Congress on Tuberculosis, Dr. John S. Fulton, the Secretary General, reported that ten distinguished foreigners have consented to participate in the series of special addresses that are to form a part of the program. The names of these eminent specialists follow: Dr. R. W. Philip, Edinburgh; Dr. C. Theodore Williams, London; Dr. Arthur Newsholme, Health Officer, Brighton, England; Dr. C. H. Spronck, Utrecht, Holland; Dr. Karl Turban, Davos-Platz, Switzerland; Dr. Gotthold Pannwitz, Charlottenburg; Dr. Emil von Behring, Marburg; Dr. A. Calmette, Pasteur Institute, Lisle, France; Dr. Maurice Letulle, Paris, and Dr. S. Kitasato, Tokyo, Japan.

Dr. Fulton also reported that, up to the date of the meeting, the Governors of twenty-three States had lent official auspices to the Congress. This not only insures official representation so far as that many States are concerned, but it insures an active organization in each of these States, that will be interested in the Congress.

The States in which this action has been taken, so far, are: California, Utah, Montana, North Dakota, Minnesota, Wisconsin, Illinois, Iowa, Indiana, Michigan, Ohio, Kentucky, Kansas, Tennessee, South Carolina, North Carolina, Maryland, New York, Massachusetts, Vermont, Maine, West Virginia and Missouri.

Reporting on the formation of State committees, the Secretary General said that such committees had been appointed in nearly all of the States in the United States; that several have already organized and are earnestly at work. He reported also that replies have been received from various foreign countries in reference to the appointment of committees, and the replies indicate that the countries addressed will be represented in nearly every instance by exhibits as well as by delegates.

---

In many cases of shock, a venous infusion will more often save life than dallying with stimulants which merely, in the end, serve to tire out the heart.—*American Journal of Surgery.*

